# Disproportionality analysis of adverse events associated with endothelin receptor antagonists based on the FDA adverse event reporting system (FAERS)

**DOI:** 10.3389/fcvm.2026.1712783

**Published:** 2026-03-10

**Authors:** Lizhu Han, Sitian Li, Jianting Liao, Qinan Yin, Changli He, Yin Wang, Gang Li, Yuan Bian

**Affiliations:** Department of Pharmacy, Personalized Drug Research and Therapy Key Laboratory of Sichuan Province, Sichuan Provincial People’s Hospital, School of Medicine, University of Electronic Science and Technology of China, Chengdu, China

**Keywords:** adverse drug events, disproportionality analysis, endothelin receptor antagonists, pulmonary arterial hypertension, signal detection

## Abstract

**Background:**

Endothelin receptor antagonists (ERAs), including bosentan, ambrisentan, and macitentan, are recognized as first-line treatments for pulmonary arterial hypertension (PAH). Although their therapeutic efficacy is well established, variations in receptor selectivity and metabolic pathways may lead to distinct adverse drug event (ADE) profiles. Nonetheless, large-scale, real-world comparative safety data remain limited. As part of routine pharmacovigilance activities, we analyzed data from the U.S. Food and Drug Administration's Adverse Event Reporting System (FAERS) to identify disproportionate reporting signals.

**Objective:**

This study leveraged data from FAERS to identify and compare potential ADEs associated with bosentan, ambrisentan, and macitentan, thereby facilitating safer and more informed clinical decision-making in pharmacotherapy.

**Methods:**

A disproportionality analysis of individual case safety reports (ICSRs) from the FAERS was performed for the period spanning Q1 2004 to Q2 2025. The classification of ADEs was conducted utilizing the Medical Dictionary for Regulatory Activities (MedDRA®) terminology. Signal detection was executed employing four distinct algorithms: the Reporting Odds Ratio (ROR), the Proportional Reporting Ratio (PRR), the Bayesian Confidence Propagation Neural Network (BCPNN), and the Multi-item Gamma Poisson Shrinker (MGPS), all based on an observational and retrospective proportional imbalance analysis of the FAERS database. Additionally, a Time-to-Onset (TTO) analysis was conducted to evaluate the temporal distribution of ADEs.

**Results:**

A total of 35,112, 48,411, and 29,877 ADE reports were collected for bosentan, ambrisentan, and macitentan, respectively. The majority of reports originated from female patients, with a male-to-female ratio of 1:2.91, aged between 18 and 65 years. Ambrisentan was disproportionally more frequently reported the highest incidence of ADEs, accounting for 42.69% of cases. Significant ADE signals were identified in the hepatobiliary, hematologic, cardiovascular, respiratory, and fluid retention-related categories. The observed ADEs predominantly comprised peripheral edema, dyspnea, liver function abnormalities, and anemia, consistent with the documented drug labeling. It is crucial that novel signals were identified, including jaw pain, pulmonary thrombosis, gout, decreased blood potassium, and hypotension. TTO analysis revealed that the majority of ADEs occurred within the first year of treatment.

**Conclusion:**

This study corroborated the established ADEs associated with ERAs and identified novel ADE signals, thereby providing new insights into their safety profile. The findings emphasize the importance of comprehensive monitoring of hepatic function, blood pressure, renal function, and electrolyte levels during ERA treatment. These results provide valuable insights for clinicians aiming to optimize ERA utilization, minimize associated risks, and improve patient outcomes. Future research should focus on elucidating the underlying mechanisms of these ADEs to further enhance the safety and efficacy of ERA therapies.

## 1 Introduction

Pulmonary Arterial Hypertension (PAH) is a severe and progressive disorder characterized by increased pulmonary vascular resistance and elevated arterial pressure, which lead to right ventricular overload, heart failure, and heightened mortality (1). The pathogenesis of PAH involves multiple interrelated pathways, with Endothelin-1 (ET-1)—a potent vasoconstrictor—playing a pivotal role. ET-1 exerts its effects through binding to endothelin receptors (ETRs), thereby promoting vasoconstriction, vascular remodeling, and inflammatory responses that contribute to disease progression (2, 3).

Endothelin comprises three primary isoforms: ET-1, Endothelin-2 (ET-2), and Endothelin-3 (ET-3). These isoforms exhibit a high degree of structural homology, each consisting of 21 amino acids, however they differ in their biological functions and distribution. The physiological effects of endothelin are mediated through its interaction with endothelin receptors, which are G-protein-coupled receptors located on the cell membrane and play a crucial role in facilitating the biological activities of the endothelin family. There are two primary subtypes: endothelin receptor A(ETA) and endothelin receptor B(ETB) (4). These receptor subtypes exhibit differences in their structural characteristics, distribution patterns, and functional roles. Bosentan and macitentan are dual ETA/ETB receptor antagonists, whereas ambrisentan is a selective ETA receptor antagonist. ETA receptors are responsible for inducing smooth muscle contraction through the facilitation of calcium ion influx, as well as stimulating the proliferation of smooth muscle cells and the development of fibrosis. These processes are critically implicated in PAH and cardiovascular diseases (5). Given their significant roles in vasoconstriction and fibrosis, a variety of therapeutic interventions for PAH, such as ambrisentan, is formulated to selectively inhibit ETA receptors, thereby reducing vascular resistance and mitigating vascular thickening and fibrosis (6). In contrast, ETB receptors are predominantly located on endothelial cells, although they are also present in certain smooth muscle cells, neurons, and renal tissues. The functional role of ETB receptors is notably intricate. They facilitate the release of nitric oxide (NO) and prostacyclin (PGI₂), thereby inducing vasodilation and diminishing vascular resistance. Additionally, they expedite the degradation of endothelin, thereby preventing its excessive accumulation (7).

In recent years, the extensive utilization of Endothelin ERAs in PAH patients has raised safety concerns that have increasingly attracted clinical scrutiny. Common ADEs associated with ERAs encompass abnormal liver function indicators, peripheral edema, anemia, nausea, and headaches (8). Furthermore, there have been documented instances of severe ADEs, including significant liver damage (9) and hemolytic anemia (10). The ERAs commonly employed in clinical practice include bosentan, ambrisentan, and macitentan. Considering the variability in ADE profiles among these medications, the selection of the most appropriate treatment for patients predisposed to such ADEs has emerged as a critical clinical challenge. Furthermore, the expanded clinical application of ERAs has led to the identification of additional serious ADEs in recent years. Consequently, further investigation and assessment of their safety profiles are imperative. Although previous studies have investigated ERA-related liver injury, anemia, and other adverse events through the FAERS, there remains a lack of cross-sectional comparisons of signal intensities among the three major types of ERAs across different time periods and diverse populations. Moreover, the reliability of emerging signals, including mandibular pain, pulmonary thrombosis, and hypokalemia, has not been systematically validated. Guideline-based pharmacovigilance studies are still missing a head-to-head comparison of the three ERAs across the same time-window and population. We therefore hypothesized that their ADE profiles differ and that some signals remain unlisted in the current labels. The objective of this study was to identify and compare potential ADEs of bosentan, ambrisentan and macitentan by mining the FAERS database, and to uncover new safety signals that might refine clinical decisions.

## 2 Materials and methods

### 2.1 Data sources

The study design and reporting followed the Reporting A Disproportionality Study Using Spontaneous Pharmacovigilance READUS-PV guidelines (Supplementary File 1). FAERS individual-case-safety-reports (ICSRs) from Q1 2004 to Q2 2025 were retrieved and de-duplicated according to FDA 2022 guidance. The original data, supplied in American Standard Code for Information Interchange (ASCII) format, were imported into My Structured Query Language (MySQL) version 15.0 and processed using Navicat Premium 15 software (11). Although the FAERS database is accessible in both Comma-Separated Values (CSV) and Extensible Markup Language (XML) formats, this study employed the CSV format due to its superior compatibility. The CSV files comprise seven datasets: Demographic Information (DEMO), Adverse Event Records (REAC), Drug Information (DRUG), Treatment Outcomes (OUTC), Report Sources (RPSR), Treatment Dates (THER), and Indication Records (INDI). To eliminate duplicate entries, we adhered to the deduplication guidelines set forth by the FDA. The process involved sorting all reports based on the Case Identifier (CASEID), which serves as a unique case identifier, and FDA Date (FDA_DT), the date of receipt by the FDA. In instances where multiple records shared the same CASEID, only the entry with the most recent FDA_DT was retained, ensuring that the most current version of the report was used. Meanwhile, records with unique CASEID values were preserved in their entirety. This procedure was conducted in accordance with the FDA's 2022 deduplication guidance, which includes considerations for follow-up reports or amendments, thereby maintaining the independence of each case for subsequent analysis.

### 2.2 Identification of adverse events and medications

In this study, drug nomenclature was standardized using Medex_UIMA_1.8.3, whereby all brand names and synonyms were mapped to their corresponding International Nonproprietary Names (INNs). Adverse events were coded according to MedDRA® version 24.0 Preferred Terms (PTs) and analyzed at both the PT and System Organ Class (SOC) levels. The route of administration was recorded when specified; in cases where it was not reported, oral administration was assumed for bosentan, ambrisentan, and macitentan. In the FAERS database, drugs are classified as Primary Suspect (PS), Secondary Suspect (SS), Concomitant (C), or Interaction (I); to enhance analytical specificity, only records in which one of the three target drugs was designated as PS were included. Throughout this manuscript, the term “ADE” refers to any adverse event documented in the database, regardless of causality attribution. No formal individual-case causality assessments [e.g., World Health Organization–Uppsala Monitoring Centre (WHO-UMC) or Naranjo scales] were conducted, and the analysis is strictly observational in nature.

### 2.3 Data processing

To detect ADE signals, four algorithms were employed, each providing unique advantages. First, the ROR is particularly effective for identifying ADEs with low reporting frequencies. Second, the PRR is characterized by high specificity, making it suitable for detailed analyses. Third, BCPNN is adept at integrating multi-source data and performing cross-validation, thereby enhancing reliability. Fourth, MGPS is ideal for detecting signals associated with rare events. These methods were selected for their complementary strengths, which facilitated an expanded the detection range and improved the reliability of the results. To enhance the robustness of signal identification and minimize false positives, a positive signal of association between a drug and an adverse event was defined only when it was concurrently detected by at least two of the four algorithms. The signal thresholds were ROR-95% confidence interva (CI) lower limit > 1, PRR-95% CI > 1, Information Component Lower Bound of 95% CI (IC025) > 0 (BCPNN) and Empirical Bayes Geometric Mean Lower Bound of 95% CI (EB05) > 0 (MGPS) (11). This study employed the full dataset comparator (12), considering all other drugs in the FAERS database except the target drugs (bosentan, ambrisentan, macitentan) as background references to construct 2 × 2 contingency tables (Table 1) and calculate the signal strength for each algorithm. The specific formulas and thresholds are detailed in Table 2. Statistical analyses were performed using Microsoft Excel 2022. Signal strength was quantified, with higher values indicating stronger correlations between the target drugs and adverse events.

**Table 1:** Four grid table of adverse reaction signal of proportional imbalance method.

Adverse drug event (ADE) type	ERAs	Non—ERAs	Total
ERAs related ADEs	a	b	a + b
Non- ERAs related ADEs	c	d	c + d
Total	a + c	b + d	a + b + c + d

**Table 2:** Calculation formula and threshold.

Method	Formula	Threshold
ROR	$ROR=\frac{a/c}{b/d}=\frac{ad}{bc}$ $95\text{\%}CI=e^{\ln(ROR)\pm 1.96\sqrt{\left(\frac{1}{a}+\frac{1}{b}+\frac{1}{c}+\frac{1}{d}\right)}}$	ROR≥3 and 95%CI > 1, (95%CI: lower limit)
PRR	PRR=[a(c+d)]/[c(a+b)] $SE(lnPRR)=\sqrt{\frac{1}{a}-\frac{1}{a+b}+\frac{1}{c}-\frac{1}{c+d}}$ $95\text{\%}CI=e^{\ln(PRR)\pm 1.96\sqrt{\left(\frac{1}{a}-\frac{1}{a+b}+\frac{1}{c}-\frac{1}{c+d}\right)}}$	PRR≥2 and 95%CI > 1, (95%CI: lower limit)
BPCNN	$IC=log_{2}\frac{P(x,y)}{P(x)P(y)}=log_{2}\frac{a(a+b+c+d)}{\left(a+b)(a+c\right)}$ $E(IC)=log_{2}\frac{\left(a+y11)(a+b+c+d+\alpha )(a+b+c+d+\beta \right)}{\left(a+b+c+d+\gamma )(a+b+\alpha 1)(a+c+\beta 1\right)}$ $V(\mathrm{IC})=\frac{1}{{\left(ln2\right)}^{2}}\left\{\left[\frac{(a+b+c+d)-\alpha +\gamma -\gamma 11}{\left(a+\gamma 11)(1+a+b+c+d+\gamma \right)}\right]+\left[\frac{(a+b+c+d)-(a+b)+\alpha -\alpha 1}{\left(a+b+\alpha 1)(1+a++c+d+\alpha \right)}\right]+\left[\frac{(a+b+c+d)-(a+c)+\beta -\beta 1}{\left(a+c+\beta 1)(1+a+b+c+d+\beta \right)}\right]\right\}$ $\gamma =\gamma 11\frac{\left(a+b++d+\alpha )(a+b+c+d+\beta \right)}{\left(a+b+\alpha 1)(a+c+\beta 1\right)}$ $IC-2SD=E(IC)-2\sqrt{V(IC)}$	IC025 > 0
EBGM	$EBGM=\frac{a(a+b+c+d)}{\left(a+c)(a+b\right)}$ $95\text{\%}CI=e^{\ln\;(EBGM)\pm 1.96\sqrt{\left(\frac{1}{a}+\frac{1}{b}+\frac{1}{c}+\frac{1}{d}\right)}}$	EBGM05 > 2

### 2.4 Data visualization

Heat-maps were generated with GraphPad Prism using the ROR as the metric; the colour scale ‘Plasma’ indicates increasing signal strength. The increase in color intensity corresponds to a higher ROR value, indicating a stronger statistical association signal between the drug and the adverse event category. Statistical analyses were performed with Microsoft Excel 2022 and GraphPad Prism version (10.1.2).

## 3 Results

This study collected a total of 35,112, 48,411, and 29,877 ADEs reports for bosentan, ambrisentan, and macitentan, respectively, as presented in Table 3, identifying these drugs as the primary suspects. In all figures and tables, bosentan is abbreviated as “bos”, ambrisentan as “amb”, and macitentan as “mac”.

**Table 3:** The fundamental information of ERA ADE reports.

Variable	bos total	amb total	mac total
Age_yr	64.00 (47.00,74.00)	63.00 (51.00,73.00)	65.00 (53.00,74.00)
Age_yrQ			
<18	2,252 (6.41)	1,087 (2.25)	312 (1.04)
18∼65	9,043 (25.75)	18,430 (38.07)	11,177 (37.41)
>=65	10,423 (29.69)	16,268 (33.60)	12,279 (41.10)
Unknown	13,394 (38.15)	12,626 (26.08)	6,109 (20.45)
Reporter			
Physician	16,142 (45.97)	9,096 (18.79)	9,729 (32.56)
Other health-professional	8,741 (24.89)	4,579 (9.46)	7,593 (25.41)
Pharmacist	5,714 (16.27)	10,843 (22.40)	10,801 (36.15)
Consumer	3,913 (11.14)	22,667 (46.82)	1,620 (5.42)
Unknown	583 (1.66)	1,225 (2.53)	91 (0.30)
Registered nurse	19 (0.05)	1 (0.00)	43 (0.14)
Outcomes			
Hospitalization	17,666 (44.25)	21,670 (45.22)	18,004 (56.69)
Death	13,688 (34.29)	7,631 (15.92)	7,208 (22.70)
Other serious	6,840 (17.13)	17,748 (37.03)	5,607 (17.65)
Life threatening	936 (2.34)	460 (0.96)	487 (1.53)
Disability	439 (1.10)	341 (0.71)	417 (1.31)
Required intervention to prevent permanent impairment/damage	331 (0.83)	59 (0.12)	9 (0.03)
Congenital anomaly	19 (0.05)	14 (0.03)	28 (0.09)
Reported countries			
United States	18,663 (53.15)	41,195 (85.09)	25,205 (84.36)
Brazil	109 (0.31)		
United Kingdom		52 (0.11)	56 (0.19)
Austria	83 (0.24)		56 (0.19)
Taiwan	77 (0.22)		107 (0.36)
Hungary	72 (0.21)		51 (0.17)
Puerto Rico			63 (0.21)
Germany	71 (0.20)	54 (0.11)	225 (0.75)
Russia	70 (0.20)		
Poland	64 (0.18)		61 (0.20)
Spain	57 (0.16)		71 (0.24)
Mexico			52 (0.17)
Turkey	56 (0.16)		
Korea, South	52 (0.15)		133 (0.45)
Other	13,860 (39.47)	5,661 (11.69)	443 (1.48)
Japan	477 (1.36)	430 (0.89)	1,530 (5.12)
Canada	387 (1.10)	739 (1.53)	456 (1.53)
Netherlands	320 (0.91)		324 (1.08)
China	221 (0.63)		
France	180 (0.51)	82 (0.17)	
Argentina		69 (0.14)	163 (0.55)
Australia	151 (0.43)	74 (0.15)	510 (1.71)
Colombia	142 (0.40)	55 (0.11)	371 (1.24)
Route			
Oral	34,332 (97.78)	27,313 (56.41)	29,683 (99.35)
Other	718 (2.04)	21,102 (43.59)	195 (0.65)
Oropharingeal	51 (0.15)		
Transplacental	12 (0.03)		
Sex			
Female	17,211 (49.02)	35,958 (74.28)	21,819 (73.03)
Male	6,221 (17.72)	11,786 (24.35)	7,732 (25.88)
Unknown	11,680 (33.26)	667 (1.38)	326 (1.09)
tto	407.00 (87.00,1,019.50)	253.00 (34.00,831.00)	178.00 (35.00,583.00)
ttoQ			
0–31	1,780 (7.87)	3,712 (8.83)	2,214 (9.88)
31–61	879 (3.89)	1,027 (2.44)	812 (3.62)
61–91	618 (2.73)	695 (1.65)	534 (2.38)
91–121	465 (2.06)	535 (1.27)	458 (2.04)
121–150	374 (1.65)	499 (1.19)	373 (1.66)
151–181	360 (1.59)	418 (0.99)	324 (1.45)
181–361	1,558 (6.89)	1,864 (4.44)	1,276 (5.69)
>=361	6,708 (29.67)	6,733 (16.02)	3,393 (15.14)
Unknown	9,864 (43.63)	26,535 (63.15)	13,031 (58.14)
wt	62.00 (48.00,80.00)	73.49 (59.02,90.72)	73.92 (59.86,90.70)
Year			
2004	581 (1.65)		
2005	339 (0.97)		
2006	288 (0.82)		
2007	215 (0.61)	54 (0.11)	
2008	298 (0.85)	477 (0.99)	
2009	371 (1.06)	815 (1.68)	
2010	5,320 (15.15)	1,445 (2.98)	
2011	3,493 (9.95)	1,438 (2.97)	
2012	3,596 (10.24)	1,796 (3.71)	
2013	3,191 (9.09)	2,776 (5.73)	
2014	2,854 (8.13)	2,766 (5.71)	1,012 (3.39)
2015	2,711 (7.72)	14,759 (30.49)	2,563 (8.58)
2016	1,796 (5.12)	2,287 (4.72)	2,686 (8.99)
2017	1,587 (4.52)	2,447 (5.05)	2,944 (9.85)
2018	1,446 (4.12)	2,605 (5.38)	3,471 (11.62)
2019	1,585 (4.51)	2,723 (5.62)	4,284 (14.34)
2020	1,811 (5.16)	2,136 (4.41)	5,667 (18.97)
2021	1,344 (3.83)	2,214 (4.57)	1,411 (4.72)
2022	939 (2.67)	2,286 (4.72)	2,033 (6.80)
2023	1,006 (2.87)	2,351 (4.86)	1,722 (5.76)
2024	246 (0.70)	2,345 (4.84)	1,450 (4.85)
2025	95 (0.27)	691 (1.43)	634 (2.12)

### 3.1 Basic information of ADE reports

**Figure 1:**
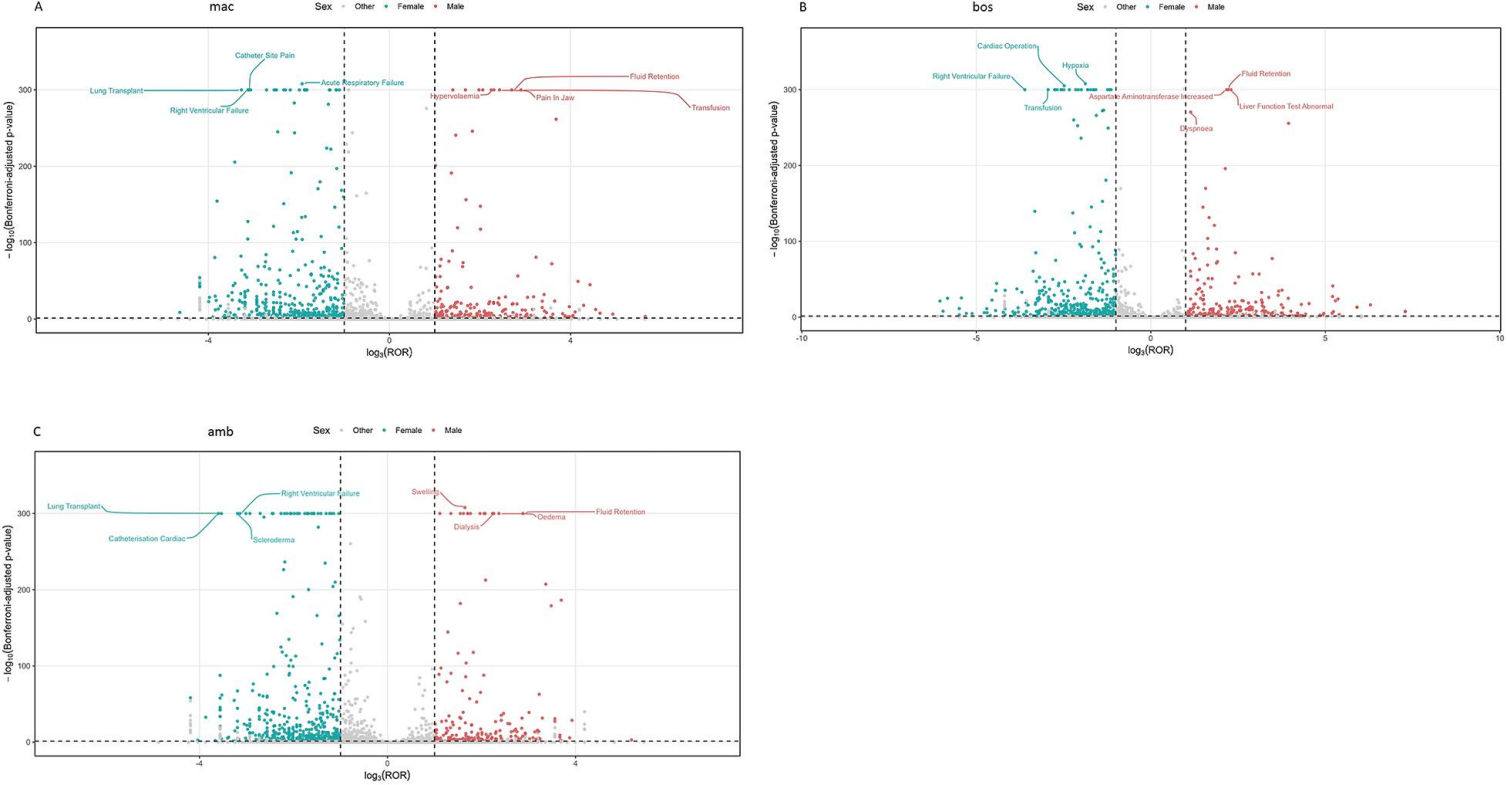
The annual distribution of ADEs associated with ERAs. **(A)** Macitentan; **(B)** bosentan; **(C)** ambrisentan. Each point represents an adverse event; the x-axis shows the log10(ROR) and the y-axis shows the -log10(*p*-value).

In terms of ADE report frequency, ambrisentan demonstrated the highest incidence at 42.69%, while macitentan exhibited the lowest incidence at 26.35%. Figure 1 illustrates the annual distribution of ADE reports for each drug. The study encompassed data from 56 countries, with the United States contributing the majority of reports: 53.15% for bosentan, 85.09% for ambrisentan, and 84.36% for macitentan. Notably, a substantial proportion of ambrisentan ADE reports were submitted by consumers (46.82%), in contrast to healthcare professionals, whereas consumer reports constituted a smaller fraction for bosentan (11.14%) and macitentan (5.42%). Furthermore, the study revealed that the number of female patients in the ADE reports significantly exceeded that of male patients across all three drugs. The male-to-female ratios in the ADE reports were 1:2.77 for bosentan, 1:3.05 for ambrisentan, and 1:2.82 for macitentan. This trend may be attributed to a higher submission rate of ADE reports by female patients and could also be influenced by the greater prevalence of PAH in females compared to males (13). Initially, PAH was classified by the National Institutes of Health (NIH) as a rare disease predominantly affecting young women, and subsequent studies have consistently shown a significantly higher proportion of female patients compared to male patients (14, 15). In terms of age demographics, the majority of ADEs associated with bosentan and macitentan were observed in patients aged 18–65 years. Conversely, among reports with available age information, ambrisentan was disproportionally more frequently reported the highest incidence of ADEs in individuals over 65 years of age. The distribution of patients aged 18–65 in the ADE reports was as follows: bosentan at 23.40%, ambrisentan at 47.68%, and macitentan at 28.92%. Despite some missing age data in the ADE reports, preliminary results suggest that the occurrence of ADEs in patients aged 18–65 is comparable to those over 65, indicating that aging does not necessarily increase the likelihood of ADEs for ERAs. Regarding clinical outcomes, hospitalization emerged as the most prevalent outcome for all three drugs, excluding cases with unspecified ADEs. The study also recorded the serious ADEs associated with three ERA drugs, specifically noting occurrences of death (13,688, 7,631, and 7,208, respectively), life-threatening outcomes (936, 460, and 487), disability (439, 341, and 417), and permanent damage (331, 59, and 9). Quantitatively, bosentan was associated with the highest number of serious ADEs, totaling 15,394 cases, followed by ambrisentan with 8,491 cases, and macitentan with 8,121 cases. Comprehensive statistics on clinical features are presented in Table 3. Additionally, the study identified a significant surge in the number of ADE reports associated with ambrisentan in 2015, as illustrated in Figure 1. This escalation may be attributed to the IA-class recommendation of ambrisentan, both as a standalone treatment and in combination with tadalafil in clinical guidelines during that period, which facilitated its widespread clinical adoption (16). In preceding years, ADE reports for the three drugs were relatively balanced. However, beginning in 2015, there was a notable decline in bosentan ADE reports compared to those for ambrisentan and macitentan. This reduction in bosentan ADE reports is not indicative of a decrease in ADE occurrences but is rather a consequence of ambrisentan and macitentan gaining greater market share, which consequently diminished the usage of bosentan.

### 3.2 ADE risk signal mining at the SOC level

In this study, ADEs associated with ERAs were classified according to the SOC outlined in version 24.0 of MedDRA® and subsequently analyzed. A total of 25 SOCs were identified as being affected by ERA-related ADEs. Table 4 delineates the signal strengths of ERA-related ADEs at the SOC level, as determined by various computational algorithms. The analysis revealed that the ADE signals for the three ERAs were predominantly concentrated within three primary SOC categories across all algorithms: “surgical and medical procedures” (bos: *n* = 6,587; amb: *n* = 4,431; mac: *n* = 8,610), “respiratory, thoracic, and mediastinal disorders” (bos: *n* = 16,553; amb: *n* = 21,571; mac: *n* = 23,304), and “cardiac disorders” (bos: *n* = 7,322; amb: *n* = 6,620; mac: *n* = 7,134). These findings are consistent with the pharmacological profile of ERAs as targeted therapies for PAH patients. Additionally, the study identified the SOC categories of “hepatobiliary disorders” (bos: *n* = 1,770; amb: *n* = 835; mac: *n* = 1,172), “metabolism and nutrition disorders” (bos: *n* = 2,804; amb: *n* = 3,860; mac: *n* = 4,767), and “blood and lymphatic system disorders” (bos: *n* = 1,523; amb: *n* = 1,415; mac: *n* = 1,974) as frequently affected by ERA-related ADEs, corroborating the information provided in the drug labeling. Interestingly, the study also identified positive ADE signals in the SOC categories of “renal and urinary disorders” (bos: *n* = 1,437; amb: *n* = 1,492; mac: *n* = 2,311), “musculoskeletal and connective tissue disorders” (bos: *n* = 3,150; amb: *n* = 4,218; mac: *n* = 6,462), and “ear and labyrinth disorders” (bos: *n* = 275; amb: *n* = 499; mac: *n* = 675), which were not previously documented in the drug labels. Comprehensive information regarding other SOC-related ADE signals is available in Table 4.

**Table 4:** The signal strength of ERA ADEs at the SOC level in FAERS database.

SOC	bos case reports	ROR(95% CI)	PRR(95% CI)	*χ*2	IC(IC025)	EBGM(EBGM05)	amb case reports	ROR(95% CI)	PRR(95% CI)	χ2	IC(IC025)	EBGM(EBGM05)	mac case reports	ROR(95% CI)	PRR(95% CI)	χ2	IC(IC025)	EBGM(EBGM05)
Surgical and medical procedures	6,587	4.28 (4.17, 4.39)	4.09 (4.01, 4.17)	15,475.98	2.02 (1.99)	4.07 (3.98)	4,431	2.65 (2.57, 2.73)	2.59 (2.54, 2.64)	4,353.46	1.37 (1.32)	2.58 (2.51)	8,610	4.03 (3.94, 4.12)	3.86 (3.79, 3.94)	18,236.59	1.93 (1.9)	3.82 (3.75)
Respiratory, thoracic and mediastinal disorders	16,553	3.29 (3.23, 3.34)	2.96 (2.9, 3.02)	22,417.98	1.56 (1.54)	2.95 (2.91)	21,571	4.28 (4.22, 4.35)	3.69 (3.62, 3.76)	44,200.17	1.88 (1.86)	3.67 (3.63)	23,304	3.65 (3.6, 3.7)	3.24 (3.18, 3.3)	37,510.56	1.69 (1.67)	3.22 (3.18)
Cardiac disorders	7,322	2.46 (2.4, 2.52)	2.37 (2.32, 2.42)	5,909.85	1.24 (1.2)	2.36 (2.31)	6,620	2.23 (2.18, 2.29)	2.17 (2.13, 2.21)	4,246.59	1.11 (1.08)	2.16 (2.12)	7,134	2.13 (2.08, 2.19)	2.08 (2.04, 2.12)	4,067.53	1.05 (1.02)	2.07 (2.03)
Investigations	12,012	1.73 (1.7, 1.77)	1.66 (1.63, 1.69)	3,322.17	0.73 (0.7)	1.65 (1.63)	6,938	0.94 (0.92, 0.96)	0.94 (0.92, 0.96)	25.11	−0.08 (−0.12)	0.94 (0.92)	11,976	1.36 (1.34, 1.39)	1.33 (1.3, 1.36)	1,054.04	0.41 (0.39)	1.33 (1.31)
Hepatobiliary disorders	1,770	1.64 (1.57, 1.72)	1.63 (1.57, 1.7)	435.08	0.7 (0.64)	1.63 (1.57)	835	0.75 (0.7, 0.8)	0.75 (0.71, 0.8)	70.08	−0.41 (−0.51)	0.75 (0.71)	1,172	0.9 (0.85, 0.95)	0.9 (0.85, 0.95)	13.74	−0.16 (−0.24)	0.9 (0.86)
Infections and infestations	9,579	1.59 (1.56, 1.62)	1.54 (1.51, 1.57)	1,918.52	0.62 (0.59)	1.54 (1.51)	10,464	1.65 (1.61, 1.68)	1.59 (1.56, 1.62)	2,410.13	0.67 (0.64)	1.59 (1.56)	11,708	1.42 (1.4, 1.45)	1.39 (1.36, 1.42)	1,362.08	0.48 (0.45)	1.39 (1.37)
General disorders and administration site conditions	23,545	1.18 (1.17, 1.2)	1.15 (1.13, 1.17)	538.2	0.2 (0.18)	1.15 (1.13)	24,368	1.15 (1.13, 1.17)	1.12 (1.1, 1.14)	373.24	0.16 (0.14)	1.12 (1.11)	28,659	1.04 (1.03, 1.05)	1.03 (1.01, 1.05)	34.65	0.05 (0.03)	1.03 (1.02)
Vascular disorders	2,927	1.16 (1.12, 1.2)	1.15 (1.11, 1.2)	61.94	0.21 (0.15)	1.15 (1.12)	3,326	1.28 (1.24, 1.33)	1.28 (1.23, 1.33)	202.46	0.35 (0.3)	1.28 (1.24)	4,123	1.35 (1.31, 1.39)	1.34 (1.29, 1.39)	360.03	0.42 (0.38)	1.34 (1.3)
Metabolism and nutrition disorders	2,804	1.11 (1.07, 1.15)	1.11 (1.07, 1.15)	29.75	0.15 (0.09)	1.11 (1.07)	3,860	1.49 (1.44, 1.53)	1.47 (1.41, 1.53)	591.02	0.55 (0.51)	1.47 (1.43)	4,767	1.5 (1.46, 1.55)	1.49 (1.46, 1.52)	773.19	0.57 (0.53)	1.48 (1.45)
Blood and lymphatic system disorders	1,523	0.75 (0.71, 0.79)	0.75 (0.71, 0.8)	123.19	−0.4 (−0.48)	0.76 (0.72)	1,415	0.67 (0.64, 0.71)	0.68 (0.64, 0.72)	219.88	−0.56 (−0.64)	0.68 (0.65)	1,974	0.76 (0.73, 0.8)	0.77 (0.74, 0.8)	143.81	−0.38 (−0.45)	0.77 (0.74)
Gastrointestinal disorders	7,539	0.73 (0.71, 0.75)	0.75 (0.74, 0.76)	709.6	−0.42 (−0.45)	0.75 (0.73)	8,609	0.8 (0.78, 0.82)	0.81 (0.79, 0.83)	404.25	−0.3 (−0.33)	0.81 (0.8)	13,325	1.03 (1.01, 1.04)	1.02 (1, 1.04)	7.68	0.03 (0.01)	1.02 (1.01)
Pregnancy, puerperium and perinatal conditions	358	0.71 (0.64, 0.79)	0.71 (0.64, 0.78)	41.2	−0.49 (−0.64)	0.71 (0.65)	84	0.16 (0.13, 0.2)	0.16 (0.13, 0.2)	359.29	−2.61 (−2.91)	0.16 (0.14)	70	0.12 (0.09, 0.15)	0.12 (0.09, 0.15)	473.14	−3.1 (−3.44)	0.12 (0.1)
Congenital, familial and genetic disorders	249	0.7 (0.61, 0.79)	0.7 (0.62, 0.79)	33.05	−0.52 (−0.7)	0.7 (0.63)	167	0.45 (0.38, 0.52)	0.45 (0.38, 0.53)	114.85	−1.16 (−1.38)	0.45 (0.39)	160	0.37 (0.32, 0.43)	0.37 (0.32, 0.43)	170.9	−1.43 (−1.65)	0.37 (0.33)
renal and urinary disorders	1,437	0.66 (0.63, 0.7)	0.67 (0.63, 0.71)	243.63	−0.58 (−0.66)	0.67 (0.64)	1,492	0.65 (0.62, 0.69)	0.66 (0.62, 0.7)	267.88	−0.6 (−0.67)	0.66 (0.63)	2,311	0.79 (0.75, 0.82)	0.79 (0.76, 0.82)	133.47	−0.34 (−0.4)	0.79 (0.76)
Nervous system disorders	6,315	0.61 (0.6, 0.63)	0.64 (0.63, 0.65)	1,448.59	−0.65 (−0.69)	0.64 (0.62)	8,949	0.86 (0.84, 0.88)	0.87 (0.85, 0.89)	193.18	−0.2 (−0.23)	0.87 (0.85)	10,823	0.87 (0.85, 0.89)	0.88 (0.86, 0.9)	197.2	−0.19 (−0.21)	0.88 (0.86)
Ear and labyrinth disorders	275	0.54 (0.48, 0.61)	0.54 (0.48, 0.61)	106.57	−0.88 (−1.05)	0.54 (0.49)	499	0.94 (0.86, 1.03)	0.94 (0.87, 1.02)	1.9	−0.09 (−0.22)	0.94 (0.87)	675	1 (0.93, 1.08)	1 (0.92, 1.08)	0.02	0.01 (−0.1)	1 (0.94)
Musculoskeletal and connective tissue disorders	3,150	0.49 (0.48, 0.51)	0.51 (0.49, 0.53)	1,593.22	−0.98 (−1.03)	0.51 (0.49)	4,218	0.63 (0.61, 0.65)	0.64 (0.62, 0.67)	887.55	−0.64 (−0.68)	0.64 (0.63)	6,462	0.79 (0.77, 0.81)	0.8 (0.78, 0.82)	359.12	−0.33 (−0.37)	0.8 (0.78)
Endocrine disorders	135	0.44 (0.38, 0.53)	0.45 (0.38, 0.54)	93.44	−1.17 (−1.41)	0.45 (0.39)	93	0.29 (0.24, 0.36)	0.29 (0.24, 0.35)	160.57	−1.78 (−2.07)	0.29 (0.25)	175	0.43 (0.37, 0.5)	0.43 (0.37, 0.5)	130.89	−1.21 (−1.42)	0.43 (0.38)
Injury, poisoning and procedural complications	5,083	0.42 (0.41, 0.44)	0.45 (0.44, 0.46)	3,788.04	−1.15 (−1.19)	0.45 (0.44)	5,616	0.43 (0.42, 0.44)	0.46 (0.45, 0.47)	3,968.08	−1.12 (−1.16)	0.46 (0.45)	5,277	0.28 (0.28, 0.29)	0.31 (0.3, 0.32)	9,174.97	−1.69 (−1.73)	0.31 (0.3)
Neoplasms benign, malignant and unspecified (incl cysts and polyps)	1,160	0.37 (0.35, 0.39)	0.38 (0.36, 0.4)	1,239.42	−1.41 (−1.5)	0.38 (0.36)	970	0.29 (0.27, 0.31)	0.29 (0.27, 0.31)	1,689.75	−1.76 (−1.85)	0.29 (0.28)	1,124	0.25 (0.23, 0.26)	0.25 (0.24, 0.27)	2,529.01	−1.97 (−2.06)	0.25 (0.24)
Immune system disorders	470	0.35 (0.32, 0.39)	0.36 (0.33, 0.4)	551.87	−1.49 (−1.62)	0.36 (0.33)	621	0.44 (0.41, 0.48)	0.44 (0.41, 0.48)	439.19	−1.17 (−1.29)	0.44 (0.42)	676	0.36 (0.34, 0.39)	0.36 (0.33, 0.39)	756.62	−1.45 (−1.56)	0.37 (0.34)
Eye disorders	701	0.29 (0.27, 0.31)	0.29 (0.27, 0.31)	1,219.56	−1.77 (−1.88)	0.29 (0.28)	1,187	0.47 (0.44, 0.49)	0.47 (0.44, 0.5)	716.3	−1.08 (−1.16)	0.47 (0.45)	1,302	0.41 (0.39, 0.43)	0.42 (0.4, 0.45)	1,088.29	−1.26 (−1.34)	0.42 (0.4)
Skin and subcutaneous tissue disorders	1,474	0.22 (0.21, 0.23)	0.23 (0.22, 0.24)	4,012.88	−2.12 (−2.19)	0.23 (0.22)	1,924	0.27 (0.26, 0.28)	0.28 (0.27, 0.29)	3,698.1	−1.82 (−1.88)	0.28 (0.27)	2,491	0.27 (0.26, 0.28)	0.28 (0.27, 0.29)	4,782.34	−1.81 (−1.87)	0.28 (0.27)
Psychiatric disorders	1,491	0.21 (0.2, 0.23)	0.22 (0.21, 0.23)	4,250.59	−2.16 (−2.23)	0.22 (0.22)	1,856	0.26 (0.25, 0.27)	0.27 (0.26, 0.28)	3,912.17	−1.89 (−1.96)	0.27 (0.26)	2,757	0.32 (0.31, 0.33)	0.33 (0.32, 0.34)	3,869.39	−1.58 (−1.63)	0.33 (0.32)
Reproductive system and breast disorders	204	0.21(0.18, 0.24)	0.21(0.18, 0.24)	604.7	−2.24 (−2.44)	0.21 (0.19)	249	0.24 (0.22, 0.28)	0.25 (0.22, 0.28)	579.42	−2.02 (−2.2)	0.25 (0.22)	338	0.29 (0.26, 0.32)	0.29 (0.26, 0.32)	602.16	−1.8 (−1.95)	0.29 (0.26)

**Figure 2:**
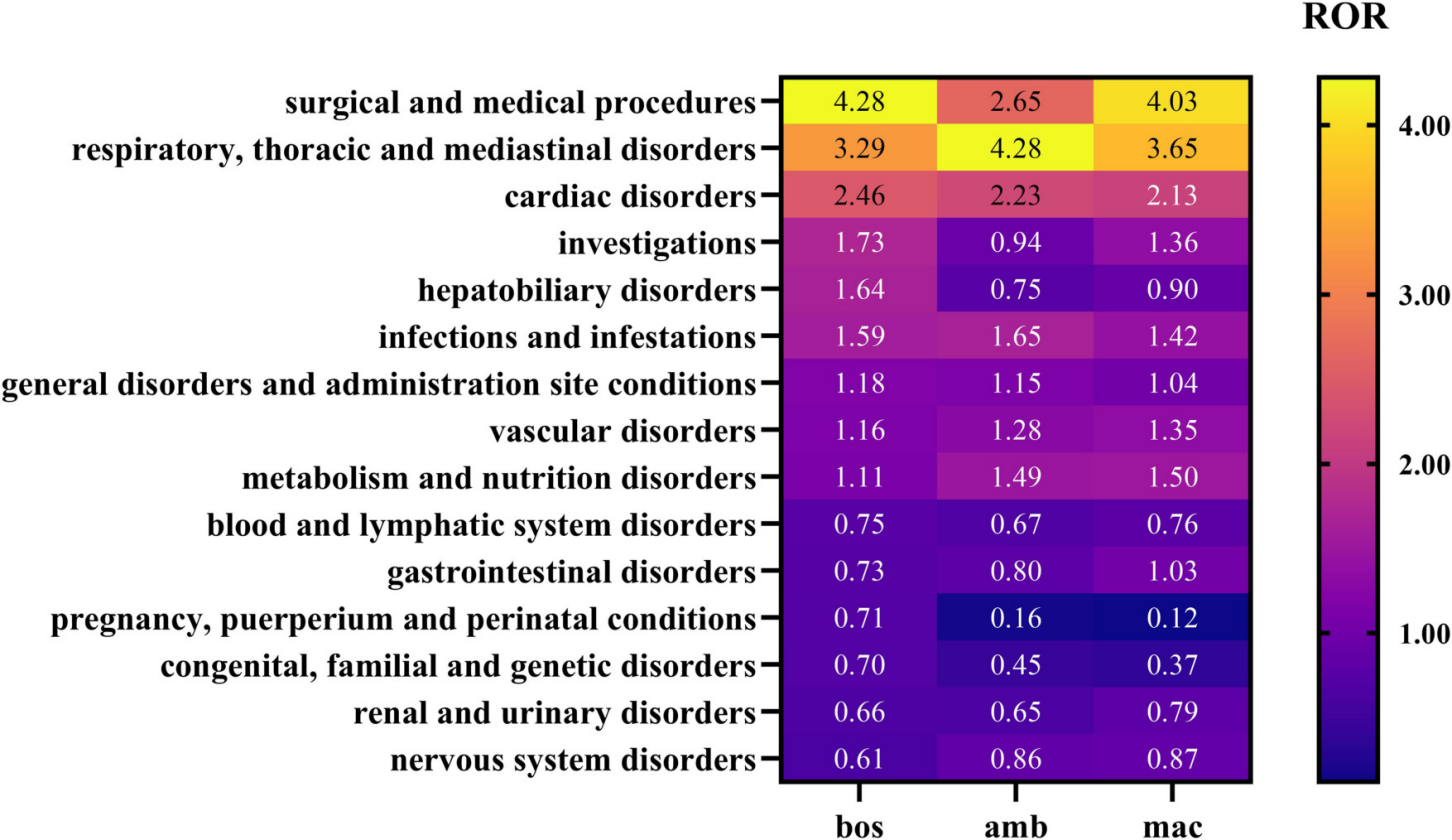
Heatmap visualization of adverse event signal strengths for bosentan, ambrisentan, and macitentan at the System Organ Class (SOC) level.

The comparative signal strengths across the three ERAs at the SOC level are visually summarized in the heatmap (Figure 2), which provides the SOC-level ROR heat-map for bosentan (bos), ambrisentan (amb) and macitentan (mac); deeper color indicates stronger signal. Notably, all three drugs exhibited substantial signals in the “Respiratory, thoracic and mediastinal disorders” and “Cardiac disorders” categories, which correspond to the pathophysiology of the underlying disease (PAH) and the mechanisms of action of the drugs.

### 3.3 ADE risk signal mining at the PT level

At the PT level, four distinct algorithms were used to analyze ADEs associated with the drugs, assessing their compliance with various screening criteria. A total of 372 valid PTs for ERAs were identified. The top 100 PTs, ranked by the total number of ADE reports for the three ERAs, are presented in Table 5. Additional positive signals are provided in Supplementary Table S1. The data indicate that the most frequently reported ADEs for the ERA drugs include “dyspnoea” (bos: *n* = 4,226; amb: *n* = 7,339; mac: *n* = 6,160), “pneumonia” (bos: *n* = 1,975; amb: *n* = 3,131; mac: *n* = 2,829), and “fluid retention” (bos: *n* = 1,037; amb: *n* = 2,157; mac: *n* = 2,071). In addition to the ADEs already documented in the drug labels, such as “abnormal liver function test”, “fluid retention” and “decreased haemoglobin”, the study identified significant positive ADE signals for terms including “pain in jaw” (bos: *n* = 200; amb: *n* = 338; mac: *n* = 702), “pulmonary thrombosis” (bos: *n* = 0; amb: *n* = 87; mac: *n* = 0), “haemoptysis” (bos: *n* = 234; amb: *n* = 236; mac: *n* = 241), “gout” (bos: *n* = 112; amb: *n* = 0; mac: *n* = 152), “increased blood bilirubin” (bos: *n* = 509; amb: *n* = 0; mac: *n* = 250), “atrial fibrillation” (bos: *n* = 632; amb: *n* = 0; mac: *n* = 0), and “decreased blood potassium” (bos: *n* = 0; amb: *n* = 0; mac: *n* = 241), which should be given special attention. Additionally, the study examined the distribution of ADE signals at the PT level across different age groups, with comprehensive details available in Supplementary Table S2. The PT ADE signals for each drug were further analyzed by gender, with detailed information available in Supplementary Table S3. The three Supplementary Tables S1–S3) contain more initial and detailed data for readers’ reference.

**Table 5:** The number of PTS listed in the top 100 in the FAERS database.

SOC	PT	bos case reports	ROR(95% CI)	PRR(95% CI)	χ2	IC(IC025)	EBGM(EBGM05)	amb case reports	ROR(95% CI)	PRR(95% CI)	χ2	IC(IC025)	EBGM(EBGM05)	mac case reports	ROR(95% CI)	PRR(95% CI)	χ2	IC(IC025)	EBGM(EBGM05)	Total
Respiratory, thoracic and mediastinal disorders	Dyspnoea	4,226	3.98 (3.86, 4.11)	3.87 (3.72, 4.02)	9,019.4	1.94 (1.9)	3.85 (3.75)	7,339	6.82 (6.66, 6.99)	6.47 (6.34, 6.6)	33,748	2.68 (2.64)	6.39 (6.26)	6,160	4.56 (4.45, 4.68)	4.42 (4.33, 4.51)	16,179.04	2.13 (2.09)	4.36 (4.27)	17,725
Infections and infestations	Pneumonia	1,975	3.23 (3.09, 3.38)	3.2 (3.08, 3.33)	2,975.56	1.67 (1.61)	3.18 (3.06)	3,131	4.91 (4.74, 5.09)	4.81 (4.63, 5)	9,393.26	2.25 (2.2)	4.77 (4.63)	2,829	3.5 (3.37, 3.63)	3.45 (3.32, 3.59)	4,891.14	1.77 (1.72)	3.42 (3.32)	7,935
Metabolism and nutrition disorders	Fluid retention	1,037	10.75 (10.1, 11.43)	10.66 (10.05, 11.31)	8,889.03	3.39 (3.3)	10.45 (9.92)	2,157	21.84 (20.9, 22.81)	21.46 (20.64, 22.32)	40,137.78	4.36 (4.29)	20.5 (19.77)	2,071	16.29 (15.58, 17.03)	16.08 (15.46, 16.72)	27,740.42	3.93 (3.87)	15.27 (14.71)	5,265
Respiratory, thoracic and mediastinal disorders	Pulmonary arterial hypertension	1,634	64.75 (61.48, 68.2)	63.84 (60.19, 67.71)	89,238.47	5.82 (5.74)	56.47 (54.07)	1,446	51.04 (48.32, 53.91)	50.44 (47.56, 53.49)	62,806.83	5.5 (5.42)	45.3 (43.27)	1,720	49.05 (46.59, 51.63)	48.5 (45.73, 51.44)	68,373.17	5.38 (5.3)	41.58 (39.83)	4,800
General disorders and administration site conditions	Oedema peripheral	943	3.93 (3.68, 4.19)	3.9 (3.68, 4.14)	2,024.73	1.96 (1.86)	3.88 (3.68)	1,841	7.91 (7.55, 8.28)	7.8 (7.5, 8.11)	10,746.24	2.94 (2.87)	7.68 (7.39)	1,415	6.58 (6.24, 6.94)	6.53 (6.16, 6.93)	6,485.12	2.68 (2.6)	6.4 (6.13)	4,199
Respiratory, thoracic and mediastinal disorders	Pulmonary hypertension	976	24.96 (23.4, 26.63)	24.76 (23.35, 26.26)	21,168.47	4.56 (4.47)	23.59 (22.35)	1,297	33.45 (31.6, 35.4)	33.1 (31.21, 35.1)	37,532.43	4.95 (4.86)	30.83 (29.4)	1,109	25.18 (23.68, 26.78)	25 (23.57, 26.51)	23,496.54	4.53 (4.44)	23.06 (21.9)	3,382
General disorders and administration site conditions	Oedema	603	5.85 (5.4, 6.34)	5.82 (5.38, 6.29)	2,382.78	2.53 (2.41)	5.77 (5.39)	1,599	15.58 (14.82, 16.38)	15.39 (14.51, 16.32)	20,792.13	3.9 (3.82)	14.89 (14.28)	1,064	8.52 (8.01, 9.06)	8.47 (7.99, 8.98)	6,810.37	3.04 (2.96)	8.25 (7.84)	3,266
General disorders and administration site conditions	Peripheral swelling	0	0	0	0	0	0	1,741	5.47 (5.22, 5.74)	5.41 (5.2, 5.63)	6,195.61	2.42 (2.35)	5.35 (5.15)	1,472	3 (2.85, 3.16)	2.98 (2.81, 3.16)	1,923.48	1.57 (1.49)	2.96 (2.83)	3,213
Cardiac disorders	Cardiac failure congestive	846	5.23 (4.88, 5.6)	5.2 (4.9, 5.51)	2,840.82	2.37 (2.27)	5.15 (4.87)	1,159	7.15 (6.75, 7.58)	7.09 (6.69, 7.52)	5,978.88	2.81 (2.72)	7 (6.66)	798	4.73 (4.41, 5.07)	4.71 (4.35, 5.09)	2,293.62	2.22 (2.11)	4.65 (4.38)	2,803
Nervous system disorders	Syncope	843	4.36 (4.07, 4.66)	4.33 (4.08, 4.59)	2,144.12	2.1 (2.01)	4.3 (4.06)	1,032	5.23 (4.92, 5.56)	5.19 (4.89, 5.5)	3,458.74	2.36 (2.27)	5.14 (4.89)	819	3.56 (3.33, 3.82)	3.55 (3.28, 3.84)	1,483.34	1.81 (1.72)	3.52 (3.32)	2,694
Investigations	Oxygen saturation decreased	580	5.56 (5.12, 6.03)	5.53 (5.11, 5.98)	2,131.62	2.45 (2.34)	5.48 (5.12)	862	8.01 (7.48, 8.57)	7.96 (7.51, 8.44)	5,154.88	2.97 (2.87)	7.83 (7.4)	1,189	8.42 (7.95, 8.93)	8.37 (7.89, 8.88)	7,498.67	3.03 (2.94)	8.16 (7.77)	2,631
General disorders and administration site conditions	Chest pain	0	0	0	0	0	0	1,326	3.72 (3.52, 3.93)	3.69 (3.48, 3.91)	2,584.81	1.87 (1.8)	3.67 (3.5)	1,281	3.16 (2.99, 3.34)	3.15 (2.97, 3.34)	1,859.74	1.64 (1.56)	3.12 (2.98)	2,607
Respiratory, thoracic and mediastinal disorders	Nasal congestion	0	0	0	0	0	0	1,444	12.62 (11.97, 13.3)	12.48 (11.77, 13.24)	14,836.72	3.6 (3.53)	12.16 (11.64)	1,131	7.5 (7.07, 7.96)	7.45 (7.02, 7.9)	6,159.59	2.86 (2.78)	7.28 (6.93)	2,575
Respiratory, thoracic and mediastinal disorders	Respiratory failure	816	5.79 (5.4, 6.21)	5.76 (5.33, 6.23)	3,173.59	2.51 (2.41)	5.7 (5.38)	882	6.19 (5.79, 6.61)	6.15 (5.8, 6.52)	3,753.02	2.6 (2.51)	6.08 (5.75)	690	4.23 (3.93, 4.56)	4.22 (3.9, 4.56)	1,671.15	2.06 (1.95)	4.17 (3.92)	2,388
Respiratory, thoracic and mediastinal disorders	Dyspnoea exertional	530	7.51 (6.9, 8.19)	7.48 (6.92, 8.09)	2,933.78	2.88 (2.76)	7.39 (6.87)	665	9.06 (8.39, 9.78)	9.01 (8.33, 9.74)	4,644.84	3.15 (3.03)	8.85 (8.3)	1,185	12.41 (11.71, 13.16)	12.33 (11.63, 13.08)	11,826.75	3.57 (3.48)	11.85 (11.29)	2,380
Cardiac disorders	Cardiac failure	775	5.02 (4.67, 5.39)	4.99 (4.61, 5.4)	2,450.8	2.31 (2.2)	4.95 (4.66)	785	4.94 (4.6, 5.3)	4.91 (4.54, 5.31)	2,423.06	2.28 (2.18)	4.87 (4.59)	768	4.01 (3.73, 4.31)	3.99 (3.69, 4.32)	1,701.86	1.98 (1.88)	3.95 (3.72)	2,328
Cardiac disorders	Right ventricular failure	780	62.28 (57.79, 67.12)	61.86 (57.2, 66.91)	41,380.6	5.78 (5.67)	54.92 (51.59)	619	46.82 (43.09, 50.87)	46.58 (43.07, 50.38)	24,943.17	5.4 (5.28)	42.17 (39.34)	665	43.93 (40.48, 47.67)	43.74 (40.44, 47.31)	24,071.54	5.25 (5.13)	38.04 (35.52)	2,064
Surgical and medical procedures	Hospitalisation	0	0	0	0	0	0	0	0	0	0	0	0	2,024	4.62 (4.42, 4.83)	4.57 (4.39, 4.75)	5,578.62	2.18 (2.11)	4.52 (4.35)	2,024
General disorders and administration site conditions	Unevaluable event	0	0	0	0	0	0	1,171	7.45 (7.03, 7.89)	7.39 (6.97, 7.84)	6,365.92	2.86 (2.78)	7.28 (6.93)	682	3.43 (3.18, 3.7)	3.42 (3.16, 3.7)	1,155.15	1.76 (1.65)	3.39 (3.18)	1,853
Respiratory, thoracic and mediastinal disorders	Pulmonary oedema	575	6.65 (6.13, 7.23)	6.63 (6.13, 7.17)	2,711.31	2.71 (2.59)	6.55 (6.11)	307	3.49 (3.12, 3.9)	3.48 (3.09, 3.91)	539.28	1.79 (1.63)	3.46 (3.15)	769	7.57 (7.05, 8.14)	7.54 (6.97, 8.15)	4,251.16	2.88 (2.78)	7.37 (6.94)	1,651
Vascular disorders	Hypotension	0	0	0	0	0	0	0	0	0	0	0	0	1,579	3.27 (3.11, 3.44)	3.25 (3.06, 3.45)	2,436.27	1.69 (1.62)	3.22 (3.09)	1,579
Respiratory, thoracic and mediastinal disorders	Hypoxia	499	7.48 (6.85, 8.17)	7.45 (6.75, 8.22)	2,747.12	2.88 (2.75)	7.35 (6.83)	459	6.83 (6.22, 7.49)	6.8 (6.17, 7.5)	2,239.22	2.75 (2.61)	6.72 (6.22)	617	7.51 (6.93, 8.14)	7.48 (6.92, 8.09)	3,379.51	2.87 (2.76)	7.32 (6.84)	1,575
Respiratory, thoracic and mediastinal disorders	Chronic obstructive pulmonary disease	542	5.4 (4.96, 5.88)	5.38 (4.97, 5.82)	1,911.77	2.41 (2.29)	5.33 (4.96)	508	4.8 (4.39, 5.24)	4.78 (4.42, 5.17)	1,503.49	2.24 (2.12)	4.74 (4.4)	459	3.67 (3.35, 4.03)	3.66 (3.32, 4.04)	878.29	1.86 (1.73)	3.63 (3.36)	1,509
Surgical and medical procedures	Transfusion	455	23.19 (21.1, 25.48)	23.1 (20.94, 25.48)	9,180.93	4.47 (4.33)	22.09 (20.41)	124	5.53 (4.63, 6.6)	5.53 (4.64, 6.6)	454.02	2.45 (2.2)	5.47 (4.72)	703	24.98 (23.12, 26.98)	24.86 (22.99, 26.89)	14,809.2	4.52 (4.41)	22.94 (21.51)	1,282
Investigations	Aspartate aminotransferase increased	1,251	12.35 (11.68, 13.07)	12.23 (11.53, 12.97)	12,591.41	3.58 (3.5)	11.95 (11.4)	0	0	0	0	0	0	0	0	0	0	0	0	1,251
Musculoskeletal and connective tissue disorders	Pain in jaw	200	3.6 (3.13, 4.14)	3.6 (3.14, 4.13)	372.39	1.84 (1.64)	3.58 (3.18)	338	5.86 (5.26, 6.52)	5.84 (5.29, 6.44)	1,339.43	2.53 (2.38)	5.78 (5.28)	702	10.17 (9.43, 10.97)	10.13 (9.37, 10.96)	5,580.06	3.3 (3.19)	9.82 (9.21)	1,240
Investigations	Alanine aminotransferase increased	1,225	10.43 (9.85, 11.04)	10.33 (9.74, 10.96)	10,113.4	3.34 (3.26)	10.13 (9.66)	0	0	0	0	0	0	0	0	0	0	0	0	1,225
Investigations	Liver function test abnormal	842	14.91 (13.92, 15.97)	14.81 (13.96, 15.71)	10,521.5	3.85 (3.75)	14.39 (13.59)	266	4.81 (4.26, 5.43)	4.8 (4.27, 5.4)	792.65	2.25 (2.08)	4.76 (4.3)	0	0	0	0	0	0	1,108
General disorders and administration site conditions	Swelling	0	0	0	0	0	0	980	5.19 (4.88, 5.53)	5.16 (4.87, 5.47)	3,252.64	2.35(2.26)	5.11(4.85)	0	0	0	0	0	0	980

**Figure 3:**
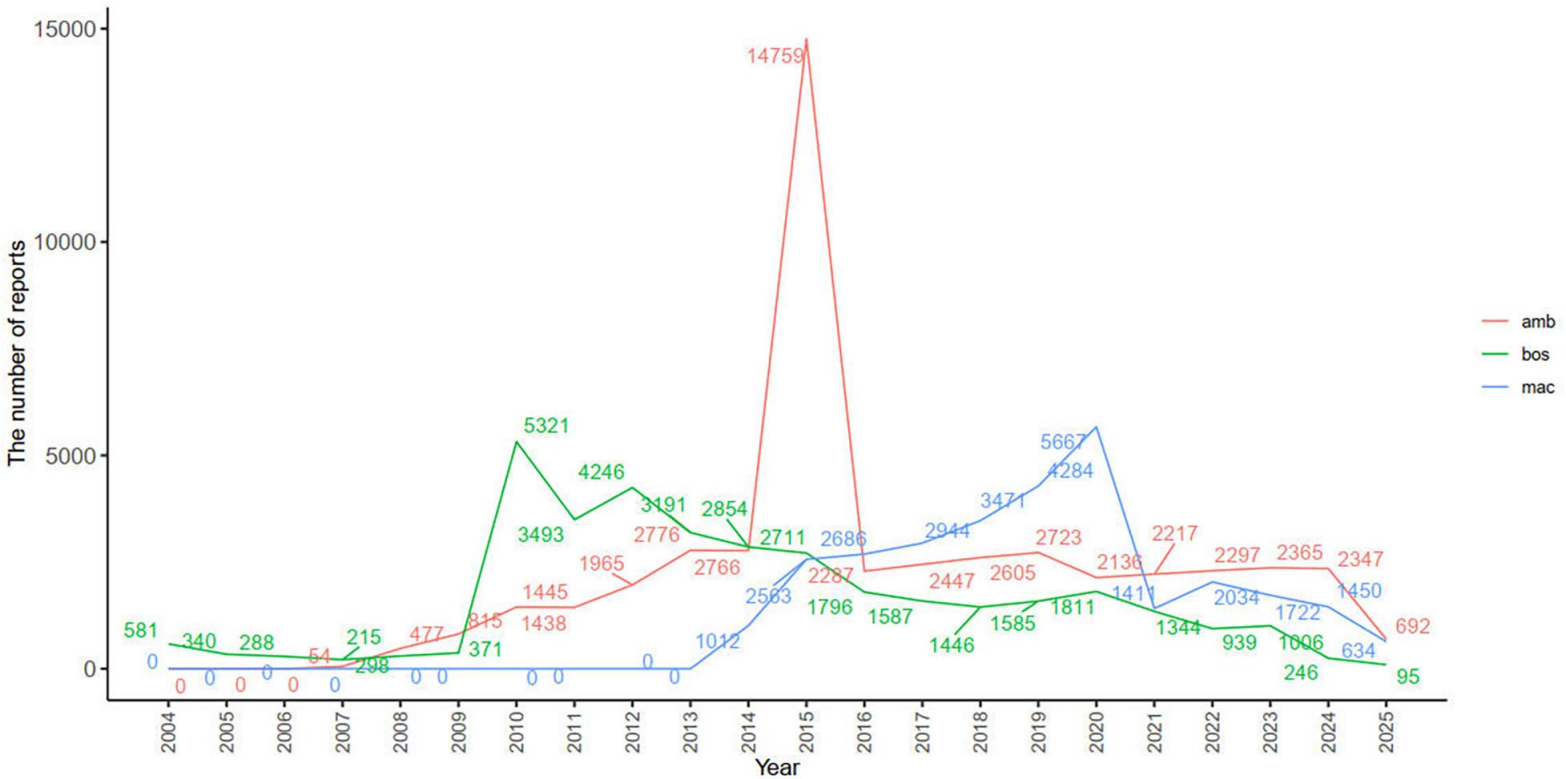
The ADE reports volcano plot for the ERAs.

Simultaneously, a visual analysis of the results was performed. According to the criteria [fold change (FC) > 1.2 and FC < 1/1.2, *P* < 0.05], the findings revealed gender-specific differences in reported ADEs among users of the three ERAs. Volcano plots (Figure 3) depict log₂-transformed female-to-male reporting odds ratio (*x*-axis) vs. –log₁₀ (*P* value) (*y*-axis). Points outside the dashed lines (*P* < 0.05 and fold-change > 1.2) denote female- or male-predominant ADEs. Take bosentan as an example. Among its users, female patients more frequently reported “cardiac operation,” “transfusion,” “hypoxia,” and “right ventricular failure” compared to male patients. Conversely, male patients more commonly reported “fluid retention” “liver function test abnormal”, “aspartate aminotransferase increased,” than female patients. These are consistent with the different physiological characteristics of estrogen receptors and the gender-specific pharmacokinetics. Further details are presented in Figure 3.

### 3.4 TTO analysis results

**Figure 4:**
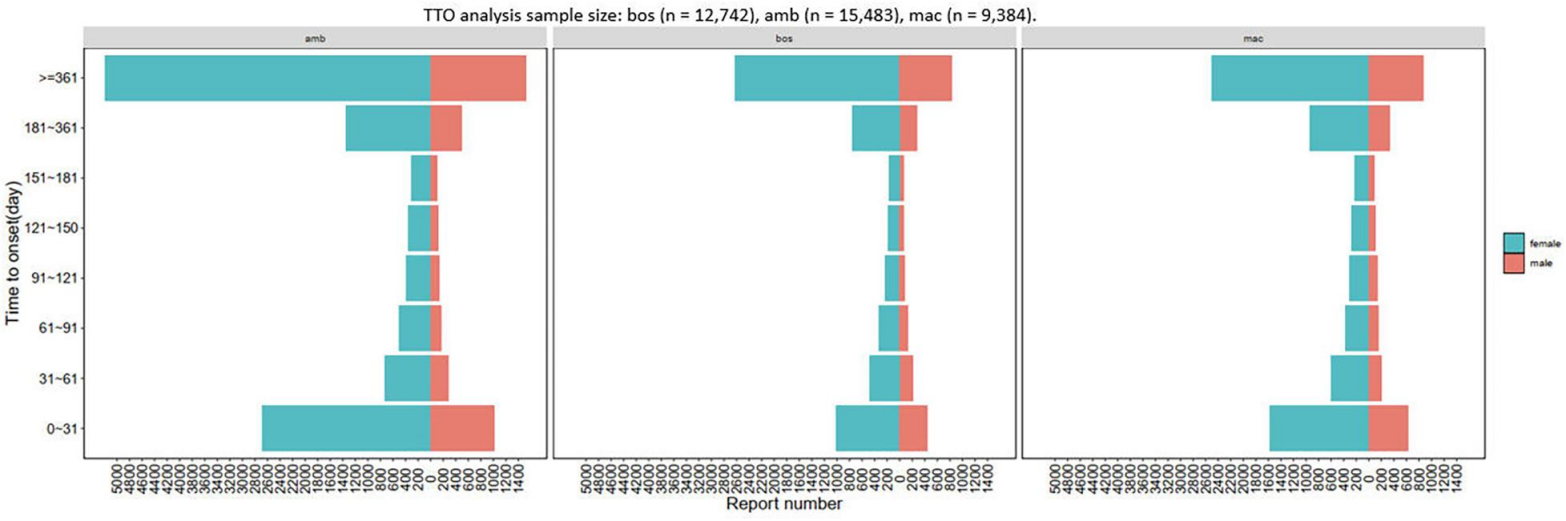
Outcomes of TTO concerning ERA ADE reports.

In the aforementioned reports involving the 372 positive PTs, datasets with comprehensive and precise records of event onset times were selected for subsequent analysis (bosentan: 12,742 reports, ambrisentan: 15,483 reports, macitentan: 9,384 reports). The subsequent phase involved conducting a Time-to-Onset (TTO) analysis of the occurrence to assess treatment-related ADEs, as illustrated in Figure 4. More than half of the flagged events (bosentan: 6,708 reports, ambrisentan: 6,733 reports; macitentan: 3,393 reports) emerged ≥12 months after initiation, underscoring the need for prolonged pharmacovigilance beyond conventional trial durations.

## 4 Discussion

ERAs serve as the primary therapeutic agents for patients with PAH and are extensively utilized in clinical settings, necessitating prolonged treatment durations. The prompt identification and mitigation of ADEs are essential for the sustained management of PAH patients. In this study, we conducted a thorough analysis of the ADEs associated with ERAs by integrating the findings of this study with existing literature. By scrutinizing the safety profiles of bosentan, macitentan, and ambrisentan, this section investigates the underlying mechanisms contributing to their differential safety outcomes and clinical implications. Key areas of focus include hepatotoxicity, fluid retention, hematologic toxicity, and other system-related ADEs. This comprehensive evaluation seeks to inform clinical decision-making and optimize the risk-benefit assessment of ERAs in the treatment of PAH and related conditions.

### 4.1 Hepatotoxicity

Hepatotoxicity is a significant and severe ADE linked to the post-marketing application of ERAs. Previous clinical studies indicate that, bosentan is most commonly associated with liver dysfunction, followed by macitentan, while ambrisentan exhibits the lowest incidence (8). The present study identified bosentan as the agent responsible for the highest number to cause the most cases of hepatobiliary disease cases (1,421 cases), with five PTs associated with hepatotoxicity: “ aspartate aminotransferase increased”, “increased alanine aminotransferase”, “increased blood bilirubin”, “abnormal liver function test” and “increased liver function test”, cumulatively accounting for 3,984 cases and demonstrating a relatively high signal strength (e.g., the ROR for “Abnormal liver function tests” is 14.91). In contrast, ambrisentan had the fewest reports of hepatobiliary diseases (371 cases), with “Abnormal liver function tests” being the associated PT (266 cases, ROR 4.81). Macitentan was associated with 506 cases of hepatobiliary diseases, with “Increased blood bilirubin” as the associated PT (250 cases, ROR 4.52).

The observed variations in hepatotoxicity among the three ERAs may be attributed to differential inhibition of the ETB receptor (17). Bosentan and macitentan are non-selective ERAs, demonstrating a marginally higher affinity for the ETA receptor relative to the ETB receptor (18, 19). In contrast, ambrisentan functions as a selective ETA receptor antagonist, exhibiting negligible affinity for the ETB receptor (20). This disparity in receptor affinity potentially influences both the therapeutic efficacy and the incidence of adverse reactions, particularly hepatotoxicity. Research indicates that the ETB receptor plays a crucial role in hepatic bile excretion. Inhibition of the ETB receptor may impair bile excretion, thereby increasing the risk of cholestasis and hepatotoxicity (21–23). Consequently, bosentan and macitentan, due to their capacity to inhibit ETB receptors, are associated with a heightened risk of hepatotoxicity, while ambrisentan, which predominantly targets the ETA receptor, is associated with a reduced risk.

Furthermore, pharmacokinetic variations exist among the three ERAs. Bosentan undergoes a complex metabolic process, predominantly via CYP3A4 and CYP2C9, resulting in numerous metabolites that can accumulate and increase hepatic burden (24, 25). Although macitentan is also a non-selective antagonist, it generates fewer metabolites and is primarily metabolized by CYP3A4, thereby imposing less hepatic strain (26). In contrast, ambrisentan is mainly metabolized by CYP3A4, yielding a limited number of low-toxicity metabolites, which reduces the risk of hepatotoxicity (27).

In summary, the differential hepatotoxicity observed among ERAs may be attributed to their receptor selectivity, metabolic pathways, and the quantity and toxicity of their metabolites. Therefore, in clinical practice, the selection of appropriate ERAs should be tailored to the patient's individual condition to minimize the risk of hepatotoxicity. For patients with hepatic dysfunction, selective ETA antagonists, such as ambrisentan, should be prioritized. Additionally, regular monitoring of liver function during ERA treatment is recommended to detect and manage potential ADEs promptly.

### 4.2 Fluid retention and peripheral edema

Fluid retention and peripheral edema are common ADEs associated with ERAs. This study identified ambrisentan as the most frequently linked to fluid retention and peripheral edema-related PTs, with a total of 10 PTs, including “peripheral edema”, “generalized edema”, and “localized edema”. These PTs accounted for 8,524 cases, comprising 2,157 instances of fluid retention (ROR = 21.84) and 1,841 instances of peripheral edema (ROR = 7.91). In comparison, bosentan was associated with a lower number of reports, totaling 2,701 cases of fluid retention and peripheral edema. Macitentan was linked to 6,174 cases related to these conditions.

The mechanisms underlying fluid retention and peripheral edema caused by ERAs are likely attributable to their multifactorial effects on the kidneys, heart, and blood vessels (4). As a selective ETA receptor antagonist, ambrisentan inhibits ETA receptors, potentially resulting in the excessive stimulation of the ETB receptor by endogenous ET-1 (28). This mechanism may activate vasopressin (AVP) and aldosterone, increasing vascular permeability and thereby causing fluid retention and peripheral edema (29). This observation elucidates why the risk of fluid retention is greater with selective ERAs compared to non-selective ERAs. Additionally, the prolonged use of ERAs may detrimentally impact renal function, resulting in sodium and water retention, and exacerbating edema symptoms.

### 4.3 Hematologic toxicity

Anemia is among the most prevalent ADEs associated with macitentan usage. This study identified that macitentan caused PTs related to anemia, including “iron deficiency anemia”, “hemoglobin decreased”, and “blood iron decreased”, with a total of 1,009 cases. In contrast, ambrisentan was associated with only 68 cases of “iron-deficiency anemia”, and no positive PTs related to anemia were observed with bosentan treatment.

A recent meta-analysis has demonstrated that the incidence of anemia in patients receiving macitentan is significantly higher compared to those in the placebo group [risk ratio (RR) = 3.86, 95% CI: 2.05–7.30] (30). Furthermore, evidence from various studies suggests that increasing doses of macitentan may lead to further reductions in hemoglobin levels (31). Although the precise mechanism underlying this effect remains unclear, it is hypothesized that fluid retention and hemodilution may play a contributory role. ERAs function by reducing pulmonary arterial pressure (PAP) through the inhibition of ET-1 and its interaction with endothelial receptors; however, this mechanism may inadvertently impact erythropoiesis, thereby elevating the risk of anemia (32). Furthermore, ERAs may alter the distribution of body fluids, resulting in a decreased the proportion of red blood cells in the circulation, which manifests as anemia (1). Anemia is a prevalent complication associated with PAH, potentially exacerbating the condition. Anemia induced by ERAs warrants particular attention, necessitating timely adjustments to targeted therapies or the administration of iron supplementation (3).

### 4.4 Respiratory and cardiovascular system-related ADEs

Previous clinical trials have identified common ADEs associated with ERAs, including dyspnea, bronchitis, syncope, and flushing (33–35). This study identified 50 PTs related to dyspnea, such as “acute respiratory failure”, “dyspnea on exertion”, and “respiratory distress”. Among these, bosentan was implicated in 11,489 cases, ambrisentan in 13,722 cases, and macitentan in 13,528 cases, with no significant differences observed among the three drugs. ERAs inhibit ETA receptors, resulting in pulmonary vasodilation and a reduction in PAP, while these physiological changes may precipitate respiratory ADEs. Firstly, the rapid dilation of pulmonary vessels can lead to a ventilation/perfusion mismatch, potentially triggering or exacerbating dyspnea and respiratory distress (36). Secondly, ET-1 plays an important role in inflammation and immune responses, and the administration of ERAs may affect the release of inflammatory mediators in the lungs, leading to symptoms such as bronchitis and coughing (37, 38). Moreover, ERAs may compromise the integrity of the alveolar-capillary membrane, leading to fluid leakage and pulmonary edema, thereby exacerbating respiratory insufficiency and potentially causing acute respiratory failure (39).

Additionally, this study identified 29 PT signals associated with cardiovascular ADEs, including “cardiac failure congestive”, “atrial fibrillation”, and “supraventricular tachycardia”. Among these, bosentan was implicated in 2,901 instances, ambrisentan in 3,301 instances, and macitentan in 1,914 instances. Notably, ambrisentan was associated with the highest number of cardiovascular ADE reports, which may be attributed to its highly selective blockade of ETA receptors. The inhibition of ETA receptors may lead to excessive activation of ETB receptors, thereby influencing the electrophysiological activity of cardiomyocytes and the function of vascular smooth muscle, ultimately elevating the risk of arrhythmias and heart failure (40).

### 4.5 Neurological system-related ADEs

Previous randomized controlled trials (RCTs) have reported ADEs related to neurological system following the administration of all three ERAs (31, 41, 42). Furthermore, post-marketing surveillance data for VOTRIGEN revealed a 9% incidence of headaches and a 7% incidence of dizziness (43). This study identified positive PTs associated with neurological system, including “syncope”, “presyncope”, “dizziness exertional”, and “dizziness postural”. Among these, 983 cases were associated with bosentan, 1,221 cases with ambrisentan, and 1,159 cases with macitentan.

ET-1 also plays a crucial role in the central nervous system by regulating cerebrovascular tone and neuronal excitability (44, 45). The blockade of ETA and/or ETB receptors by ERAs may lead to dysregulated cerebrovascular function, resulting in alterations in cerebral hemodynamics and symptoms such as headaches and dizziness. Additionally, ERAs can influence peripheral blood pressure, leading to fluctuations in blood pressure and orthostatic hypotension, thereby increasing the risk of syncope and pre-syncope (46). Exertional dizziness may be attributed to impaired blood pressure regulation during physical activity, while sinus headaches could be linked to vasodilation or the release of inflammatory mediators with the cerebral vasculature. Simultaneously, given that other PAH-targeted medications, such as riociguatd and phosphodiesterase 5 inhibitors (PDE5i), can significantly influence blood pressure, it is imperative to closely monitor peripheral blood pressure during the treatment of PAH patients to prevent hypotensive syncope (3).

### 4.6 Gastrointestinal ADEs

This study did not identify significant associations with adverse events such as nausea, abdominal pain, and diarrhea, which may imply that ERAs exert minimal effects on gastrointestinal function or that these ADEs are less frequently reported in clinical settings. Nonetheless, ERAs might induce alterations in the gastrointestinal vascular system, such as “large intestinal haemorrhage” and “ gastric antral vascular ectasia”, indicating that their vasodilatory properties could heighten the risk of gastrointestinal bleeding. ERAs function by inhibiting ETA and/or ETB receptors, leading to vasodilation and hemodynamic changes, which could enhance blood flow and permeability in the gastrointestinal mucosa, thereby increasing the risk of bleeding (47, 48).

### 4.7 New ADE positive signal

Table 6 presents a summary of the comparative analysis between the top 100 preferred terms (PTs) associated with adverse events and the FDA DailyMed labels as of 2025-01-15. A designation of “Y” indicates that the corresponding PT was not identified in any section—including black box warnings, contraindications, precautions, adverse reactions, and post-marketing information—across the product labeling of bosentan, ambrisentan, and macitentan. Furthermore, these PTs were consistently detected by all ≥ 2 signal detection algorithms employed in this study, thereby qualifying them as “new signals.” The subsequent discussion will focus on a detailed examination of representative new signals that are closely relevant to the primary topic under investigation, including “pain in jaw”, “thrombosis”, “blood potassium decreased”, and “gout”.

**Table 6:** PT-level novelty assessment against 2025-01-15 FDA DailyMed labels (top 100 PTs).

No.	Preferred term	Bosentan label	Ambrisentan label	Macitentan label	Novel (Y/N)
1	Dyspnoea	Y	Y	Y	N
2	Pneumonia	Y	Y	Y	N
3	Fluid retention	Y	Y	Y	N
4	Pulmonary arterial hypertension	Y^a^	Y^a^	Y^a^	N
5	Oedema peripheral	Y	Y	Y	N
6	Pulmonary hypertension	Y^a^	Y^a^	Y^a^	N
7	Oedema	Y	Y	Y	N
8	Peripheral swelling	Y	Y	Y	N
9	Cardiac failure congestive	Y	Y	Y	N
10	Syncope	Y	Y	Y	N
11	Oxygen saturation decreased	Y	N	N	N
12	Chest pain	Y	Y	Y	N
13	Nasal congestion	N	Y	Y	N
14	Respiratory failure	Y	Y	Y	N
15	Dyspnoea exertional	Y	Y	Y	N
16	Cardiac failure	Y	Y	Y	N
17	Right ventricular failure	Y	Y	Y	N
18	Hospitalisation	N	N	Y	N
19	Unevaluable event	N	Y	N	N
20	Pulmonary oedema	Y	Y	Y	N
21	Hypotension	N	N	Y	N
22	Hypoxia	Y	Y	Y	N
23	Chronic obstructive pulmonary disease	Y	Y	Y	N
24	Transfusion	N	N	N	Y
25	Aspartate aminotransferase increased	Y	N	N	N
26	Pain in jaw	N	N	N	Y
27	Alanine aminotransferase increased	Y	N	N	N
28	Liver function test abnormal	Y	Y	N	N
29	Swelling	Y	Y	Y	N
30	Acute respiratory failure	Y	Y	Y	N
31	Lung transplant	N	N	N	Y
32	Catheterisation cardiac	Y	Y	Y	N
33	Haemoglobin decreased	N	N	Y	N
34	Blood bilirubin increased	Y	N	Y	N
35	Disease progression	N	N	N	Y
36	Dialysis	N	N	N	Y
37	Pericardial effusion	Y	Y	Y	N
38	Device related infection	Y	Y	Y	N
39	Haemoptysis	Y	Y	Y	N
40	Blood alkaline phosphatase increased	Y	N	N	N
41	Palpitations	N	Y	N	N
42	Lung disorder	N	N	N	Y
43	Therapy interrupted	N	Y	N	N
44	Atrial fibrillation	Y	N	N	N
45	Ascites	Y	N	Y	N
46	Presyncope	Y	Y	Y	N
47	Scleroderma	Y	Y	Y	N
48	Pleural effusion	N	N	Y	N
49	Productive cough	N	N	Y	N
50	Cardiac pacemaker insertion	N	N	N	Y
51	Oxygen consumption increased	Y	Y	Y	N
52	Cardiac operation	N	N	N	Y
53	Therapy non-responder	N	N	Y	N
54	Concomitant disease aggravated	N	N	Y	N
55	Infusion site pain	N	Y	Y	N
56	Intentional dose omission	N	Y	N	N
57	Pulmonary fibrosis	Y	Y	N	N
58	Pulmonary arterial pressure increased	Y	Y	Y	N
59	Therapy change	N	Y	Y	N
60	Sinus congestion	N	Y	Y	N
61	Atrial flutter	Y	N	Y	N
62	Catheter site pain	N	N	N	Y
63	Catheter site erythema	N	N	N	Y
64	Cyanosis	N	N	N	Y
65	Exercise tolerance decreased	Y	Y	Y	N
66	Hepatic cirrhosis	Y	N	Y	N
67	Disease complication	N	N	Y	N
68	Generalised oedema	Y	Y	Y	N
69	Gout	N	N	N	Y
70	Hypervolaemia	N	Y	Y	N
71	Gamma-glutamyltransferase increased	Y	N	N	N
72	Pulmonary congestion	N	N	N	Y
73	Catheter site infection	N	N	N	Y
74	Hospice care	N	N	N	Y
75	Blood potassium decreased	N	N	N	Y
76	Respiratory distress	N	N	N	Y
77	Catheter management	N	N	N	Y
78	Brain natriuretic peptide increased	N	N	N	Y
79	Seasonal allergy	N	N	N	Y
80	Viral infection	N	N	N	Y
81	Stent placement	N	N	N	Y
82	Cardiac failure acute	Y	Y	Y	N
83	Left ventricular failure	Y	Y	Y	N
84	Infusion site erythema	N	Y	Y	N
85	Central venous catheterisation	N	Y	N	N
86	Cardiomegaly	N	N	N	Y
87	Terminal state	N	N	N	Y
88	Catheter placement	N	N	N	Y
89	Pregnancy	N	N	N	Y
90	Catheter site haemorrhage	N	N	N	Y
91	Cor pulmonale	Y	Y	N	N
92	Vascular device infection	N	Y	Y	N
93	Respiration abnormal	N	N	N	Y
94	Iron deficiency anaemia	N	N	N	Y
95	Cholecystectomy	N	N	N	Y
96	Haematocrit decreased	N	N	N	Y
97	Oxygen saturation abnormal	N	N	N	Y
98	Transplant evaluation	N	N	N	Y
99	Dyspnoea at rest	N	N	N	Y
100	Cardiac ablation	N	N	N	Y

Y = PT absent from all three FDA-approved labels and positive in ≥2 algorithms; *N* = PT already listed.

^a^ If listed under “indication” or disease description, it is not considered an adverse reaction, but still counted as “already occurred”.

Specifically, there were 200 cases of jawbone pain associated with bosentan, 338 cases linked to ambrisentan, and 702 cases related to macitentan. The underlying etiology of jaw pain may involve the effects of ERAs on vascular function and bone metabolism. ET-1 plays a pivotal role in bone remodeling by modulating the activity of osteoblasts and osteoclasts to maintain skeletal homeostasis (49, 50). By interfering with the binding of ET-1 to its receptors, endothelin receptor antagonists (ERAs) may disturb bone metabolic equilibrium, potentially resulting in decreased bone mineral density or microarchitectural deterioration, which may contribute to the pathogenesis of temporomandibular joint disorders. Notably, the biological functions of ET-1 extend beyond skeletal regulation; it has been shown to suppress insulin-stimulated cell proliferation, impair myogenic differentiation, and promote muscle atrophy through ETB receptor activation and the p38 MAPK signaling pathway (49, 50). These direct effects on musculoskeletal tissues, in conjunction with altered bone metabolism, may synergistically contribute to the development of temporomandibular joint dysfunction and associated pain.

Thrombosis-related PTs include “pulmonary congestion,” “pulmonary thrombosis,” “catheter site haemorrhage” and “thromboembolectomy.” Bosentan was implicated in 140 cases, ambrisentan in 121 cases, and macitentan in 253 cases. Given the elevated risk of thrombosis in patients with pulmonary arterial hypertension (PAH), distinguishing between thrombotic events induced by treatment and those arising from the underlying disease remains challenging. Currently, there is insufficient evidence to establish a direct causal association, such as drug-induced pulmonary thrombosis. Consequently, none of the three medications are referenced in the FDA-approved prescribing information concerning this adverse event. Thrombosis associated with ERAs may be attributed to their impacts on endothelial function and the coagulation system. The endothelin system is integral to maintaining the integrity of the vascular endothelium and ensuring an anticoagulant state (51). Inhibition of ETA and/or ETB receptors could lead to endothelial dysfunction, thereby promoting platelet aggregation and the activation of coagulation factors, which could elevate the risk of thrombosis, particularly in individuals with pre-existing risk factors, such as atherosclerosis (52).

In relation to the observed “blood potassium abnormal”, ambrisentan was implicated in 20 cases, while macitentan was implicated in 198 cases. The SERAPHIN (53) clinical study, conducted in 2013, was the first to quantitatively establish the hypokalemia adverse reaction rate associated with macitentan at 6.2%. Alterations in blood potassium levels, including hypokalemia or hyperkalemia, may be associated with the effects of ERAs on aldosterone secretion and renal tubular function. When macitentan simultaneously blocks both ETA and ETB receptors, the negative feedback regulation of endothelin-1 (ET-1) via ETB receptors in the juxtaglomerular apparatus is abolished, leading to overactivation of the renin-angiotensin-aldosterone system (RAAS). Subsequently, aldosterone upregulates epithelial sodium channels (ENaC) and renal outer medullary potassium channels (ROMK) in the distal tubule, promoting sodium reabsorption and potassium excretion (54). ERAs may indirectly stimulate aldosterone secretion, resulting in increased sodium reabsorption and potassium excretion by the renal tubules, which can potentially lead to hypokalemia. Conversely, renal dysfunction may impair potassium excretion, thereby causing hyperkalemia (55). Consequently, it is essential to monitor blood potassium levels during ERA therapy to prevent electrolyte imbalances.

Furthermore, 112 cases of gout-related PTs were reported following bosentan administration, and 152 cases were reported following macitentan administration. In 2012, the Canadian product monograph for bosentan included gout and hypokalemia as rare adverse reactions observed during post-marketing surveillance, with an incidence rate of less than 1% (56). However, gout was not officially reported by the FDA or classified as a confirmed adverse reaction for bosentan. The development of gout may be attributed to the effects of ERAs on uric acid excretion, potentially resulting in decreased uric acid clearance, elevated serum uric acid levels, and the subsequent development of gout. Moreover, renal dysfunction caused by ERAs may exacerbate the risk of hyperuricemia.

## 5 Research limitations

This study is subject to several limitations. Firstly, the FAERS database is dependent on voluntary reporting, which may result in underreporting, erroneous or incomplete submissions from non-clinical individuals, thereby compromising data quality and consistency. Secondly, the dataset is predominantly representative of Western populations, with limited representation of Asian populations, potentially limiting the generalizability of the findings across diverse ethnic groups. Thirdly, the frequent co-administration of multiple medications among patients complicates the exclusion of potential confounding effects due to drug interactions when attributing adverse drug events. Furthermore, as a disproportionality analysis, this study is subject to methodological limitations including masking, notoriety bias, and the Weber effect, all of which may affect signal detection and strength (57). For instance, the marked increase in ambrisentan reports in 2015 coincided with clinical guideline endorsements and extensive media coverage, potentially leading to heightened reporting driven by publicity rather than true risk escalation. Residual masking from drug–drug interactions or indication channelling thus remains plausible. Lastly, the absence of formal causality assessment at the individual case safety report (ICSR) level precludes definitive verification of causal relationships. Consequently, further real-world observational studies and mechanistic investigations are warranted to validate and interpret these signals.

## 6 Conclusion

Utilizing data from the FAERS data spanning from the first quarter of 2004 to the second quarter of 2025, this study systematically assessed the safety signals associated with ERAs bosentan, ambrisentan, and macitentan. The findings reaffirmed that hepatotoxicity, fluid retention, anemia, and dyspnea are consistent adverse effects across all three ERAs, with stable signal intensities observed over time. Newly identified positive signals include mandibular pain, pulmonary thrombosis, gout, hypokalemia, and macitentan-associated hypotension, which should be incorporated into clinical safety monitoring protocols. In clinical practice, it is recommended that liver function, blood pressure, hemoglobin levels, electrolytes, and uric acid be closely monitored during the first year of treatment and throughout any dose adjustment periods. For patients with a history of hepatic disease or electrolyte disturbances, preferential use of highly selective ETA antagonists is advised, along with shortened intervals for follow-up evaluations. Furthermore, we urge clinical professionals to actively report ADEs associated with ERAs observed during follow-up visits, thereby contributing valuable data for the clinical management of PAH.

## Data Availability

The datasets presented in this study can be found in online repositories. The names of the repository/repositories and accession number(s) can be found in the article/Supplementary Material.
